# Case report: bilateral optic nerve head drusen and glaucoma

**Published:** 2017

**Authors:** Mihaela-Roxana Mănoiu, Jade Amine Amri, Alexandra Țicle, Cristina Stan

**Affiliations:** * Department of Ophthalmology, Emergency District Hospital Cluj-Napoca, Romania; ** Department of Ophthalmology, “Iuliu Hațieganu” University of Medicine and Pharmacy Cluj-Napoca, Romania

**Keywords:** optic nerve head drusen, glaucoma

## Abstract

Optic nerve head drusen is an incidental finding in current ophthalmological practice. Although patients rarely display symptoms, structural, and functional defects, there are exceptional cases when clinical appearance can make it difficult to diagnose underlying or coexisting conditions, such as glaucoma.

The following case report demonstrates how overlapping optic nerve pathologies can interfere in clinical judgement and therapeutic decision making in a young male patient, with relevant family history for both glaucoma and bilateral optic nerve head drusen.

## Background 

Optic Nerve Head Drusen (ONHD) is an ancillary ophthalmological finding that describes focal, usually calcified deposits within the optic nerve, such as mucopolysaccharides and proteins, which result from axonal metabolism. These deposits are present in up to 2% of the population, with bilateral involvement in 75% of the cases [**1**,**2**]. Both sporadic occurrence and autosomal dominant inheritance have been reported [**3**]. According to literature reports, there are two variants of ONHD, known as “visible drusen” and “buried drusen”. The latter is commonly diagnosed among younger patients and can misrepresent the edges of the optic disc and cup [**3**]. This appearance changes throughout the years, as the drusen becomes more visible and starts to protrude, usually on the inferior nasal side [**3**,**4**].

Although it is often asymptomatic, ONHD exerts a crowding effect that can further lead to structural and functional ophthalmological changes, such as retinal nerve fiber layer thinning and visual field loss. On this account, glaucoma should be considered as an important part of the differential diagnosis. In addition, patients with ONHD display a family history of glaucoma more frequently compared to healthy controls [**5**]. 

## Case Report

A 17-year-old active, male patient was admitted to the eye clinic, without prior treatment or ophthalmological evaluation. Chief complaints were mild, discontinuous headaches and blurred vision on both eyes. The patient presented with no personal pathological background. Family history revealed that the patient’s father, aged 41, had been diagnosed with bilateral optic nerve head drusen, and advanced open angle glaucoma, with a severe impairment of the visual function. Furthermore, the father underwent standard trabeculectomy in both eyes and maximal medical therapy was associated in order to render the target intraocular pressure (IOP).

On hospital admission, the general physical examination was normal, whereas the ophthalmological evaluation provided both normal and peculiar findings, as it follows: 

1. Using the Snellen Chart, visual acuity testing was 20/ 20 in the right eye (uncorrected visual acuity), as well as in the left eye (best corrected visual acuity/ -0.50 spherical diopters). 

2. Ocular motility and slit lamp examination of the anterior segment were within normal range. 

3. Goldmann Applanation Tonometry demonstrated ocular hypertension, with daily variations of IOP between 20 to 24 mmHg, in the RE, and 24 to 26 mmHg, in the LE. 

4. Corneal thickness measured 554 microns in the RE, respectively 563 microns in the LE. 

5. Gonioscopy (Goldmann 3-mirror lens) indicated a Shaffer grade 4 open angle, in both eyes, with wide ciliary band. 

6. Fundus examination disclosed resembling changes in both eyes and consisted of small optic discs, that centrally displayed multiple yellow, round elevations, with ”no apparent cupping”.

Considering the clinical appearance, which was highly suggestive for ONHD, additional morphological and functional tests were performed. The aim was to provide an accurate positive and differential diagnosis of anomalous optic disc. 

**Fig. 1 a,b:**
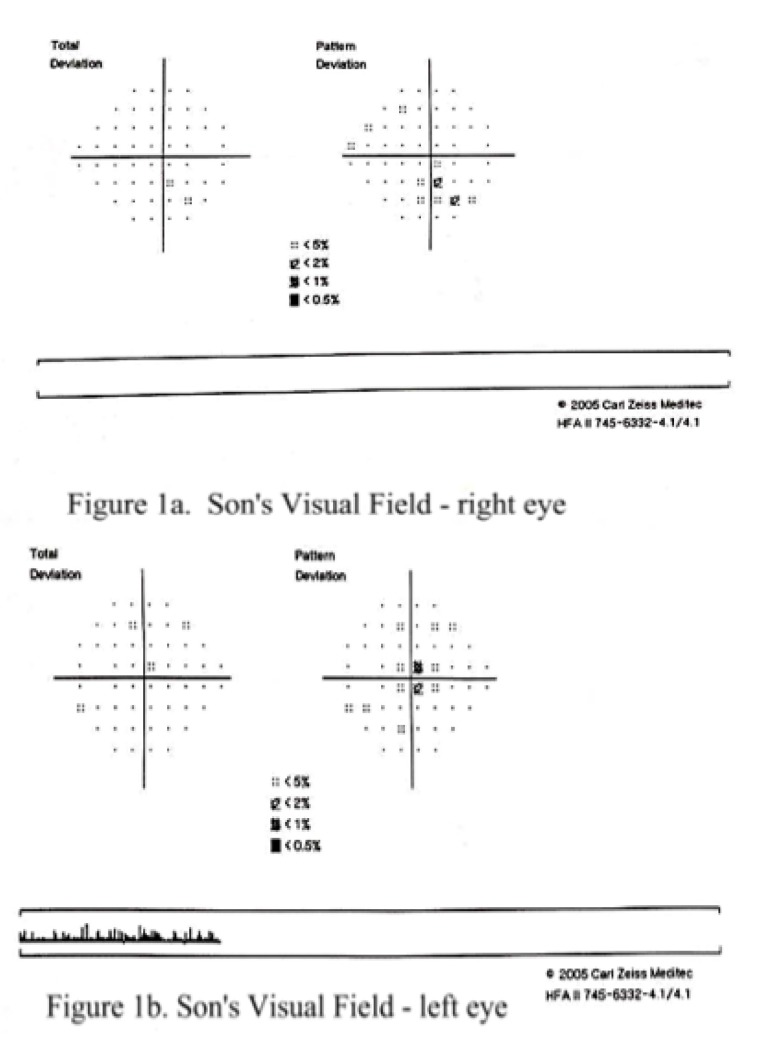
Son’s Humphrey Visual Field testing 24-2 (pattern deviation) demonstrating minimal, unspecific changes: inferior scotoma right eye; central scotoma left eye

Consequently, multiple 24-2 visual field tests, with Humphrey Field Analyser (HFA) were carried out, since the patient did not have a previous perimetric inquiry, in order to establish a proper baseline test for the future progression analysis and follow-ups. They resulted in minimal, asymmetrical changes (**Fig. 1 a,b**).

**Fig. 2:**
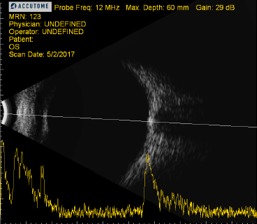
A and B-scan ocular ultrasound showing highly reflective optic nerve head drusen

In addition, ocular ultrasound (B-scan) exposed morphological changes with highly reflective drusen in both eyes, identical to the lesions found in the patient’s father (**Fig. 2**).

**Fig. 3:**
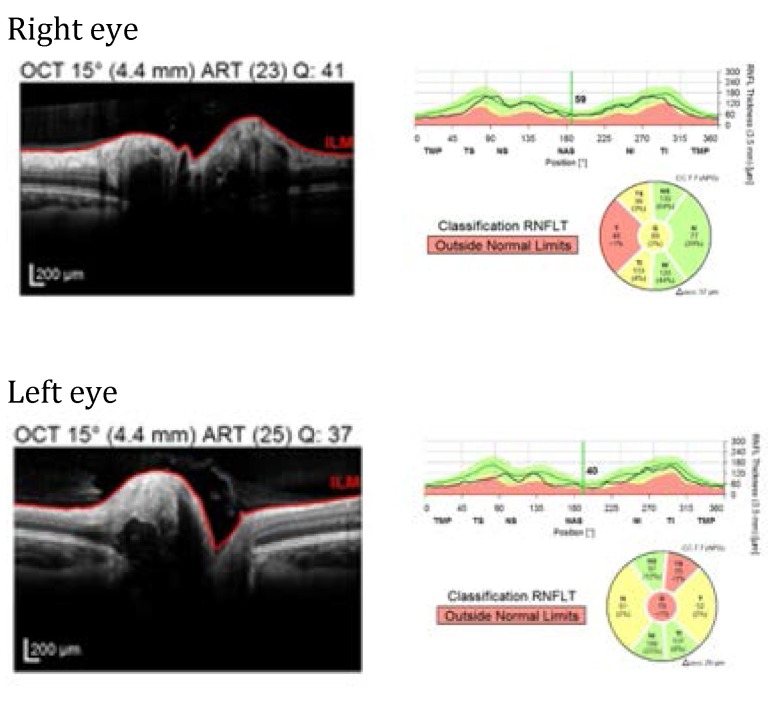
Son’s SPECTRALIS OCT, both eyes: temporal RNFL thinning; internal hyperreflective focus, external hyperreflective edge and hyporeflective area in between

Heidelberg optical coherence tomography (SPECTRALIS OCT) was indicated for a more complex evaluation of the structural changes in the retinal nerve fiber layer (RNFL) and ganglion cell layer (GCL). Both eyes exposed temporal thinning of RNFL and GCL, as well as internal hyperreflective focus, external hyperreflective edge and hyporeflective area in between (**Fig. 3**).

Given the past medical background of the patient, with relevant family history for concomitant ONHD and primary open angle glaucoma, along with the clinical ophthalmological findings, and the complementary structural and functional explorations, the case was looked upon as juvenile glaucoma, associated with ONHD. 

On the grounds of the established positive diagnosis, topical prostaglandin analogues were indicated (1 drop/ once/ daily). Target IOP level was set at 15 mmHg and the patient was referred to regular ophthalmological check-ups for further treatment adjustment. 

Currently, the patient carries out medical visits in his hometown territorial hospital, due to distance related limits. 

## Discussions

ONHD are autofluorescent, calcified deposits, which commonly occur in small, crowded optic discs [**6**]. Likewise, in the previously presented case, the clinical ophthalmological appearance corresponds to the one described in literature reviews. In terms of epidemiology, the prevalence of ONHD is reported more frequently among female population, and bilateral involvement is routinely observed [**7**,**8**]. The 17-year-old male patient and his father presented almost identical features of the optic disc, on both eyes. 

Moreover, the family history in the above-mentioned case, strongly suggested an autosomal dominant inheritance pattern. However, studies have led to inconclusive results. Lorentzen (1961), as well as Antcliff and Spalton (1999) could not validate this pattern of genetic transmission [**9**,**10**]. A prospective study showed that the family history of glaucoma was approximately 10 times more frequent in patients with ONHD in comparison to healthy patients and half as frequent as in glaucoma patients [**5**]. On this account, the young patient in the case report was thoroughly evaluated and IOP-lowering medication was considered necessary. 

Optic nerve head drusen and glaucoma are different pathological entities that can both cause similar visual-field defects [**11**]. This results in the diagnostic and therapeutical difficulties in current practice, generated by coexisting ONHD, ocular hypertension and visual field loss. According to Grippo et al., visual field defects are highly prevalent in eyes with ONHD and concomitant ocular hypertension [**12**]. Although ONHD is usually asymptomatic, the risk for visual field loss is higher for grade III ONHD (dense drusen with the optic cup obscured) compared to grade I ONHD (few scattered drusen present), with the same IOP level. Consequently, a proper IOP-lowering prevents additional visual field loss [**12**,**13**]. This data supports the therapeutical approach described previously in the case report. Lee et al. (2005) analyzed the rate of visual field loss in patients with ONHD, over a 36-months follow up. The result was 1.6% per year, with a significant damage in older patients [**14**]. Therefore, the functional outcome for the young patient in the above-mentioned case report was good. 

A particular feature that further facilitates the differentiation between glaucoma and ONHD is a significantly decreased ocular blood flow [**15**]. In recent studies, ocular blood flow analysis, using flowmetry with calculation of an individual normal range of IOP grants high efficacy in predicting coexisting glaucoma in patients with ONHD [**15**,**16**]. Nonetheless, this complementary examination was overlooked during the hospital admission of the 17-year-old patient. 

In contrast to other diagnostic tools, OCT enables early disclosure of RNFL thinning, caused by ONHD [**17**]. Evidence on this topic revealed that the prevalent site for RNFL thinning in patients with ONHD is the nasal, peripapillary region [**2**,**18**]. OCT findings often display a normal appearance in cases of buried ONHD, but RNFL thinning has been observed in all peripapillary quadrants in cases with visible drusen [**2**,**18**]. However, Gili et al. were unable to demonstrate significant thinning in the temporal quadrant; they attributed this to the less common occurrence of drusen in the temporal disc [**19**]. In contrast to these studies, the patient in the case report presented with a RNFL thinning in the temporal quadrant. In a very recent report, GCL thickness and RNFL both decreased significantly with visible optic drusen, while GCL thickness decreased more than RNFL thickness in buried drusen. The authors emphasized that GCL analysis was more sensitive than RNFL in the detection of axon damage seen with drusen [**20**].

## Conclusion 

ONHD represents an apparent trivial pathology, often overlooked in current ophthalmological practice. Despite its common asymptomatic occurrence, it can cause functional impairment and structural defects that can mimic glaucomatous neuropathy damage. Although it is usually an independent entity, ONHD can be related to or associated with other ocular conditions. For this reason, a thorough ophthalmological assessment should always be performed, in order to provide an overview on natural history, prognostic features and therapeutic management of ONHD.

**Acknowledgment**

Stan Cristina, Mănoiu Mihaela Roxana, Amri Amine Jade and Țicle Alexandra have contributed equally to this article; in consequence they have the same position as main authors in this paper.
